# Tandem Mass Spectrometry Screening for Inborn Errors of Metabolism in Newborns and High-Risk Infants in Southern China: Disease Spectrum and Genetic Characteristics in a Chinese Population

**DOI:** 10.3389/fgene.2021.631688

**Published:** 2021-07-28

**Authors:** Jianqiang Tan, Dayu Chen, Rongni Chang, Lizhen Pan, Jinling Yang, Dejian Yuan, Lihua Huang, Tizhen Yan, Haiping Ning, Jiangyan Wei, Ren Cai

**Affiliations:** Key Laboratory of Prevention and Control of Birth Defects, Department of Medical Genetics, Newborn Screening Center, Liuzhou Maternity and Child Health Care Hospital, Liuzhou Institute for Reproduction and Genetics, Affiliated Maternity Hospital and Affiliated Children’s Hospital of Guangxi University of Science and Technology, Liuzhou, China

**Keywords:** disease spectrum, genetic characteristics, genetic mutation, incidence of IEMs, inborn errors of metabolism, tandem mass spectrometry

## Abstract

Inborn errors of metabolism (IEMs) often causing progressive and irreversible neurological damage, physical and intellectual development lag or even death, and serious harm to the family and society. The screening of neonatal IEMs by tandem mass spectrometry (MS/MS) is an effective method for early diagnosis and presymptomatic treatment to prevent severe permanent sequelae and death. A total of 111,986 healthy newborns and 7,461 hospitalized high-risk infants were screened for IEMs using MS/MS to understand the characteristics of IEMs and related gene mutations in newborns and high-risk infants in Liuzhou. Positive samples were analyzed by Sanger sequencing or next-generation sequencing. The results showed that the incidence of IEMs in newborns in the Liuzhou area was 1/3,733, and the incidence of IEMs in high-risk infants was 1/393. Primary carnitine deficiency (1/9,332), phenylketonuria (1/18,664), and isovaleric acidemia (1/37,329) ranked the highest in neonates, while citrullinemia type II ranked the highest in high-risk infants (1/1,865). Further, 56 mutations of 17 IEMs-related genes were found in 49 diagnosed children. Among these, *HPD* c.941T > C, *CBS* c.1465C > T, *ACADS* c.337G > A, c.1195C > T, *ETFA* c.737G > T, *MMACHC* 1076bp deletion, *PCCB* c.132-134delGACinsAT, *IVD* c.548C > T, c.757A > G, *GCDH* c.1060G > T, and *HMGCL* c.501C > G were all unreported variants. Some related hotspot mutations were found, including *SLC22A5* c.51C > G, *PAH* c.1223G > A, *IVD* c.1208A > G, *ACADS* c.625G > A, and *GCDH* c.532G > A. These results show that the overall incidence of IEMs in the Liuzhou area is high. Hence, the scope of IEMs screening and publicity and education should be expanded for a clear diagnosis in the early stage of the disease.

## Introduction

Inborn errors of metabolism (IEMs), also known as inherited metabolic diseases, is a group of hereditary diseases caused by genetic defects, which lead to the functional defects of some enzymes, carriers, receptors, and other proteins needed to maintain normal metabolism. IEMs result in the disorder of biochemical metabolic pathways, accumulation of intermediate or bypass metabolites, or a lack of terminal metabolites. There are several forms of IEMs, including amino acid metabolism disorders (including urea cycle disorders, branched-chain amino acid metabolism abnormalities, and sulfur amino acid metabolism disorders), organic acidemia, fatty acid oxidation metabolism disorders, glucose metabolism disorders, and other diseases. Dozens of genetic metabolic diseases, such as amino acid metabolic disorders, organic acid metabolic disorders, and fatty acid oxidation disorders, can be screened for simultaneously with high flux and high accuracy by determining the amino acid and acylcarnitine content in neonatal dried blood on filter paper. This method has gradually become popular in China in recent years. A large number of genetic metabolic diseases can be detected at an early stage in newborns due to the wide popularization of tandem mass spectrometry in China. The incidence rate of IEMs varies among different countries and populations worldwide (Webster et al., 2003; Yoon et al., 2005; Sanderson et al., 2006; Dhondt, 2010; Lindner et al., 2011; Lim et al., 2014; Hassan et al., 2016; Karaceper et al., 2016; Yunus et al., 2016; Alfadhel et al., 2017; Shibata et al., 2018).

The incidence of IEMs also varies greatly among the different cities and regions within China (Guo et al., 2018; Wang et al., 2019). Significant differences in IEMs spectra, prevalence, and genetic characteristics exist among the different regions and populations in China. The Liuzhou area has 48 ethnic minorities, and the custom of intermarriage among these ethnic groups is widespread. In Liuzhou, newborn screening for congenital hypothyroidism and phenylketonuria (PKU) began 1994, screening for glucose-6-phosphate dehydrogenase deficiency, thalassemia, and congenital adrenocortical hyperplasia began in 2000, and tandem mass spectrometry screening for genetic metabolic diseases began in 2012. At present, the screening coverage rate of genetic metabolic diseases is about 50% of newborns in Liuzhou, and it is expected that the tandem mass spectrometry screening of IEMs in Liuzhou area will reach full coverage in newborns in 2025. In this study, the amino acid and acylcarnitine profiles of 111,986 newborns born in Liuzhou from December 2012 to June 2020 and 7,461 hospitalized children with suspected genetic metabolic diseases were screened, and then diagnoses were confirmed by first-generation sequencing or high-throughput sequencing. Finally, 30 cases of genetic metabolic diseases were diagnosed in 111,986 newborns, at an incidence rate of 1/3,733; 19 cases were diagnosed in high-risk infants, at an incidence rate of 1/393. Further, 56 mutations of 17 IEMs-related genes were found in 49 diagnosed children. What is noteworthy is that the incidence of primary carnitine deficiency (PCD) is the highest in Liuzhou, about 1/9,332, which is higher than that reported in other areas of China. *SLC22A5* gene c.51C > G may be a hotspot mutation in Liuzhou, but whether it is related to ethnic groups in this area needs to be further explored in the future.

## Materials and Methods

### Ethical Approval

This study was approved by the Ethical Committee of Liuzhou Maternity and Child Health Hospital, and informed consent of the parents.

### Study Cohort and Specimen Collection

From December 2012 to June 2020, The heel blood of newborns 3 days after birth and high-risk infants was collected, dripped on filter paper (Waterman Company, S&S903#, Britain), and dried naturally at room temperature. Dried blood samples from 111,986 newborns and 7,461 hospitalized children with suspected genetic metabolic diseases were collected. Among the 111,986 newborns, there were 53,753 Han, 40,763 Zhuang, 10,394 Dong, 6,217 Miao and 859 Yao. There were 5,596 Han, 4,594 Zhuang, 2,096 Zhuang, 543 Dong, 130 Miao and 98 Yao among the 7,461 high risk infants.

### Tandem Mass Spectrometry Screening

Dried blood spots were pretreated using a non-derivatized MS/MS kit according to the manufacturer instructions (FengHua, China) and then analyzed using a tandem mass spectrometry system (ABI3200, United States). The screening indicators included: free carnitine (C0); 11 amino acids and 33 acylcarnitines (Table 1). Repeated MS/MS tests were performed on the recalled positive samples, and biochemical tests or genetic analysis were performed on the re-examined positive cases.

**TABLE 1:** Blood markers and cutting values of amino acid and acyl carnitine spectrum.

**Indicators**	**Minimum value (μmol/L)**	**Maximum value (μmol/L)**
Ala	120	600
Gly	170	1,150
Pro	110	400
Leu	51	295
Val	45	270
Met	5	38
Phe	24	116
Tyr	45	260
Cit	5	41
Orn	42	390
Arg	1	52
C0	9.00	55.00
C2	8.00	50.00
C3	0.35	4.20
C3DC	0.02	0.20
C4	0.06	0.50
C4-OH	0.03	0.40
C4DC	0.05	0.50
C5	0.04	0.50
C5:1	0.00	0.10
C5-OH	0.05	0.60
C5DC	0.01	0.20
C6	0.02	0.20
C6:1	0.01	0.10
C6DC	0.00	0.10
C8	0.01	0.24
C8:1	0.04	0.45
C8DC	0.01	0.10
C10	0.02	0.25
C10:1	0.02	0.25
C12	0.03	0.60
C12:1	0.01	0.30
C14	0.07	0.50
C14:1	0.02	0.40
C14-OH	0.00	0.10
C16	0.30	5.70
C16:1	0.03	0.45
C16-OH	0.00	0.10
C18	0.14	1.86
C18:1	0.40	3.00
C18-OH	0.00	0.05
C3/C0	0.03	0.25
C3/C2	0.04	0.25
C4/C2	0.00	0.03
C5/C2	0.00	0.03
C5-OH/C3	0.02	0.40
C5DC/C8	0.20	3.00
C8/C2	0.00	0.10
C14:1/C8:1	0.10	4.00
C0/(C16 + C18)	2.00	30.00
(C16 + C18:1)/C2	0.07	0.40

### Genetic Analysis

For all positive samples, 2 mL of whole-blood samples were collected from the newborns and their parents after obtaining the informed consent of the guardian and the approval of the hospital ethics committee. Genomic DNA was extracted to amplify all exons and part of the intron region at the exon boundary. The amplified products were sequenced by Sanger sequencing and compared with the human genome gene sequence. Some genetic metabolic diseases, such as PKU and methylmalonic acidemia (MMA), which are caused by multiple genes, were screened by high-throughput sequencing by Jiajian Medicine (Guangzhou, China) and then verified by Sanger sequencing.

### Bioinformatics Analysis

The Human Gene Mutation Database,^1^ single-nucleotide polymorphism,^2^ ExAC,^3^ and 1,000 Genomes Project^4^ were queried for the newly discovered variation sites, and protein functions were predicted using Sorting Intolerant from Tolerant and PolyPhen-2 bioinformatics software. At the same time, the pathogenicity was interpreted according to the standards and guidelines for the interpretation of gene variation issued by the American Society of Medical Genetics and Genomics in 2015.

### Statistical Analysis

Statistical analysis was performed using SPSS17.0 version. The difference of categorical data was compared using Chi-square test. The difference of measurement data was compared by analysis of variance. *p* < 0.05 was considered to be statistical significance. Primary screening positive rate = number of positive cases at primary screening/number of children screened × 100%; positive predictive value = number of confirmed cases/number of positive recalls × 100%; incidence = number of confirmed cases/number of children screened × 100%.

## Results

### Screening Results of Amino Acids and Acylcarnitines in Newborns

Among the 111,986 newborns, 2,464 tested positive at the primary screening, with a positive rate of 2.2% (2,464/111,986), and 2,275 suspected positive cases were recalled, with a positive recall rate of 92.3% (2,275/2,464). Finally, 30 cases of genetic metabolic diseases were clinically diagnosed (the disease spectrum is shown in Table 2, and the MS/MS results are shown in Table 3), and the positive predictive value was 1.3%. Preliminary statistics showed that the incidence of genetic metabolic diseases in the Liuzhou area was 1/3,733. The total incidence of amino acid metabolic diseases was 1/13,998, of which there were six cases of phenylalanine hydroxylase deficiency (PHD) (1/18,664; highest), one case of hyperhomocysteinemia (1/111,986), and one case of citrullinemia type l (CTLN2; 1/111,986).

**TABLE 2:** Disease spectrum of 30 cases of genetic metabolic diseases screened from 111,986 newborns.

**Categories**	**Total**	**Incidence rate**	**IEM**	**Cases**	**Incidence rate**
AAMD	8	1/13,998	PKU	6	1/18,664
			CTLN2	1	1/111,986
			HCY	1	1/111,986
FAMD	15	1/7,466	PCD	12	1/9,332
			SCADD	2	1/55,993
			MCADD	1	1/111,986
OAMD	7	1/15,998	IVA	3	1/37,329
			MMA	1	1/111,986
			PA	1	1/111,986
			GA-1	1	1/111,986
			HMGCLD	1	1/111,986

**TABLE 3:** Results of tandem mass spectrometry in 49 children with genetic metabolic diseases (μmol/L).

**IEM**	**Cases**	**Reference result**	**Reference value**	**Preliminary screening results *M* (min∼max)**	**Re-examination results *M* (min∼max)**
PKU	6	Phe	24.0–116.0	378.3 (201–1000.5)	376.9 (230.5–789.3)
		Phe/Tyr	0.2–2.0	10.24 (3.02–25.6)	14.64 (4.77–20.12)
CTLN2	5	Cit	5–41	236.2 (128.3–365.7)	240.12 (134.8–345.7)
		Met	5–38	129.0 (94.0–181.5)	145.5 (124.5–163.2)
OTC	2	Cit	5–41	1.85 (1.6–2.1)	2.05 (1.8–2.3)
		Cit/Phe	0.14–0.72	0.095 (0.09–0.10)	0.075 (0.06–0.09)
CPS1	1	Cit	5–41	2.4	2.8
HCY	1	Met	5–38	152.6	480
		Met/Phe	0.25–1.2	2.65	3.20
H-TYR	1	Tyr	45–260	526.0	498.2
		Phe/Tyr	0.2–2.0	0.12	0.14
PCD	14	C0	9.0–55.0	2.83 (0.74–5.45)	3.95 (1.07–5.82)
SCADD	4	C4	0.06–0.50	1.99 (1.69–2.49)	2.06 (1.02–2.84)
		C4/C2	0–0.03	0.09 (0.069–0.114)	0.11 (0.062–0.169)
MCADD	1	C8	0.01–0.24	1.89	3.53
		C8/C2	0–0.1	0.19	0.42
CPT2	1	C16	0.3–5.7	24.2	28.6
		C18	0.14–1.86	4.35	5.02
		C18:1	0.30–2.9	4.65	4.36
		(C16 + C18:1)/C2	0.07–0.40	14.0	13.8
MADD	1	C4	0.06–0.50	2.59	2.64
		C5	0.04–0.50	6.98	7.32
		C6	0.01–0.10	0.62	0.56
		C8	0.01–0.24	1.24	1.12
		C10	0.01–0.30	1.70	1.58
		C12	0.02–0.30	1.92	1.56
		C14	0.05–0.40	2.44	2.68
IVA	4	C5	0.04–0.50	7.5 (5.6–11.6)	5.90 (4.32–8.96)
		C5/C2	0–0.03	0.57 (0.35–0.74)	0.66 (0.48–0.83)
GA-1	3	C5DC	0.01–0.2	1.75 (0.98–2.70)	2.34 (1.58–3.78)
		C5DC/C8	0.2–3.0	65.9 (17.5–139.9)	69.8 (26.5–120.0)
MMA	2	C3	0.35–4.2	8.83 (8.70–8.96)	11.1 (8.32–13.87)
		C3/C2	0.04–0.25	0.94 (0.56–1.32)	0.69 (0.62–0.76)
		C3/C0	0.03–0.25	1.33 (0.46–2.2)	0.56 (0.51–0.61)
PA	2	C3	0.35–4.2	9.67 (9.61–9.73)	8.30 (7.82–8.78)
		C3/C2	0.04–0.25	2.24 (1.53–2.94)	1.16 (0.85–1.46)
		C3/C0	0.03–0.25	1.57 (1.06–2.08)	1.58 (1.46–1.69)
HMGCLD	1	C5-OH	0.05–0.6	2.38	2.51
		C5-OH/C3	0.02–0.4	4.65	1.02

**FIGURE 1:**
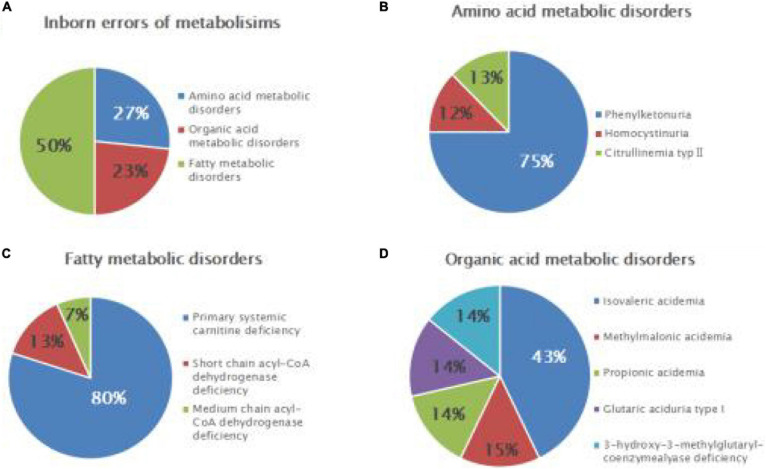
Disease spectrum and distribution of inborn errors of metabolism (IEMs) in newborns. Relative proportions of different categories of IEMs **(A)**, amino acid metabolic disorders **(B)**, fatty acid metabolic disorders **(C)**, and organic acid metabolic disorders **(D)**.

Seven cases of organic acidemia (1/15,998) were reported, of which isovaleric acidemia (IVA) was the highest (1/37,329); besides, there were one case of MMA (1/111,986), one case of glutaric acidemia type I (1/111,986), and one case of 3-hydroxy-3-methyl glutaric acidemia (1/111,986). Fifteen cases of fatty acid metabolism disorder (1/7,466) were found, of which PCD was the highest (1/9,332); besides, there were two cases of short-chain acyl-CoA dehydrogenase deficiency (SCADD; 1/55,993) and one case of medium-chain acyl-CoA dehydrogenase deficiency (MCADD; 1/111,986) (Table 2 and Figure 1).

### Diagnosis of IEMs in High-Risk Infants

**FIGURE 2:**
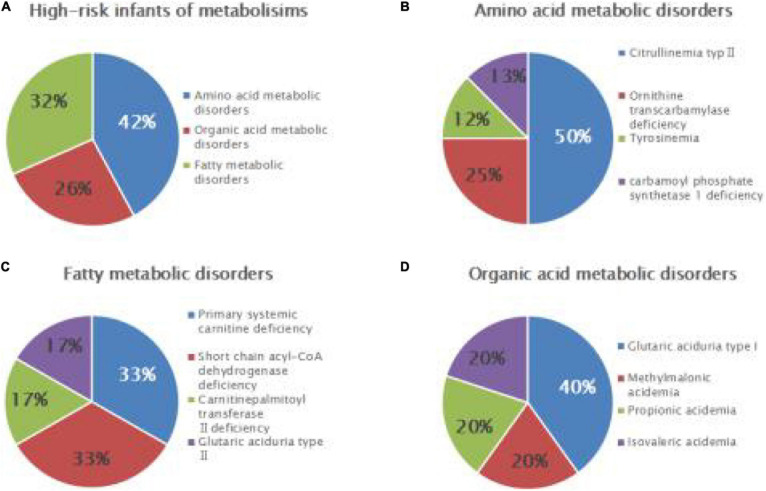
Disease spectrum and distribution of IEMs in high-risk infants. Relative proportions of different categories of IEMs **(A)**, amino acid metabolic disorders **(B)**, fatty acid metabolic disorders **(C)**, and organic acid metabolic disorders **(D)** in high-risk infants.

Among 7,461 hospitalized high-risk infants with suspected genetic metabolic diseases, 19 cases were diagnosed as genetic metabolic diseases, including eight cases of amino acid metabolic diseases (1/933), of which there were four cases of CTLN2 (1/1,865), two cases of ornithine carbamoyltransferase deficiency (1/3,731), one case of tyrosinemia (H-TYR; 1/7,461), and one case of carbamyl phosphate synthase I deficiency (CPS1; 1/7,461). Further, six cases of fatty acid metabolism disorder (1/1,244) were found, including two cases of PCD (1/3,731), two cases of SCADD (1/3,731), one case of carnitine palmitoyltransferase II deficiency (CPT2; 1/7,461), and one case of glutaric acidemia type II (1//7,461). Moreover, five cases of organic acidemia (1/1,492) were reported, including two cases of glutaric acidemia type I (1/3,731), one case of propionic acidemia (PA; 1/7,461), one case of IVA (1/7,461), and one case of MMA (1/,7461) (Table 4 and Figure 2).

**TABLE 4:** Disease spectrum of 19 children with genetic metabolic diseases screened from 7,461 high-risk infants.

**Categories**	**Total**	**Incidence rate**	**IEM**	**Cases**	**Incidence rate**
AAMD	8	1/933	CTLN2	4	1/1,865
			OTC	2	1/3,731
			CPS1	1	1/7,461
			H-TYR	1	1/7,461
FAMD	6	1/1,244	PCD	2	1/3,731
			SCADD	2	1/3,731
			CPT2	1	1/7,461
			MADD	1	1/7,461
OAMD	5	1/1,492	GA-1	2	1/3,731
			MMA	1	1/7,461
			PA	1	1/7,461
			IVA	1	1/7,461

### Gene Detection Results

The gene detection results revealed 56 mutations of 17 IEMs-related genes in 49 diagnosed children. A total of 11 kinds of 26 mutation sites were detected in the *SLC22A5* gene: c.51C > G, c.976C > T, c.1411C > T, c.1195C > T, c.760C > T, c.919delG, c.1400C > G, c.517delC, c.839C > T, c.505C > T, and c.338G > A. A total of eight kinds of 12 mutation sites were detected in the *PAH* gene: c.1174T > A, c.1223G > A, c.208_210del, c.611A > G, c.331C > T, c.28G > A, c.320A > G, and c.721C > T. Also, five mutation types and eight mutation sites were found in the *IVD* gene: c.158G > A, c.214G > A, c.548C > T, c.757A > G, and 1208A > G. The *ACADS* gene had six kinds of eight mutation sites: c.268G > A, c.320G > A, c.337G > A, c.625G > A, c.1031A > G, and c.1148G > A. A total of three kinds and six mutation sites were detected in the *GCDH* gene: c.532G > A, c.655G > A, and c.1060G > T. The detection of other disease-related genes is shown in Tables 5, 6. Among these, *HPD* gene c.941T > C, *CBS* gene c.1465C > T, *ACADS* gene c.337G > A and c.1195C > T, *ETFA* gene c.737G > T, *MMACHC* gene 1076-bp deletion, *PCCB* gene c.132-134delGACinsAT, *IVD* gene c.548C > T and c.757A > G, *GCDH* gene c.1060G > T, and *HMGCL* gene c.501C > G were all unreported variants.

**TABLE 5:** Spectrum of IEMs and variants in confirmed cases of newborns.

**IEM**	**Gender**	**Ethnicity**	**Gene**	**Variant 1**	**Variant 2**
PKU			*PAH*		
1	M	Zhuang		c.1174T > A (p.F392I)	c.1223G > A (p.R408Q)
2	M	Han		c.1223G > A (p.R408Q)	c.208_210del (p.70del)
3	F	Han		c.611A > G (p.Tyr204Cys)	c.611A > G (p.Tyr204Cys)
4	F	Han		c.331C > T (p.Arg111Ter)	c.728G > A (p.Arg243Gln)
5	M	Han		c.320A > G (p.His107Arg)	c.1223G > A (p.Arg408Gln)
6	M	Han		c.721C > T (p.Arg241Cys)	c.728G > A (p.Arg243Gln)
CTLN2			*SLC25A13*		
1	M	Yao		c.851_854delGTAT	c.1638_1660dup23
HCY			*CBS*		
1	F	Han		c.1465C > T (p.Gln489Ter)^#^	c.1465C > T (p.Gln489Ter)^#^
PCD			*SLC22A5*		
1	M	Han		c.51C > G (p.Phe17Leu)	c.760C > T (p. Arg254Ter)
2	M	Han		c.760C > T (p. Arg254Ter)	c.919delG (p.Val307LeufsX14)^#^
3	F	Han		c.1400C > G (p.Ser467Cys)	c.1400C > G (p.Ser467Cys)
4	F	Han		c.1195C > T (p.Arg399Try)	c.517delC (p.Leu173CysfsX3)
5	F	Zhuang		c.51C > G (p.Phe17Leu)	c.51C > G (p.Phe17Leu)
6	F	Han		c.51C > G (p.Phe17Leu)	c.338G > A (p.Cys113Tyr)
7	F	Han		c.1400C > G (p.Ser467Cys)	c.839C > T (p.Ser280Phe)
8	F	Miao		c.976C > T (p.Gln326X)^#^	c.505C > T (p.Arg169Trp)
9	M	Han		c.760C > T (p. Arg254Ter)	c.760C > T (p. Arg254Ter)
10	M	Han		c.51C > G (p.Phe17Leu)	c.51C > G (p.Phe17Leu)
11	M	Zhuang		c.51C > G (p.Phe17Leu)	c.51C > G (p.Phe17Leu)
12	M	Han		c.338G > A (p.Cys113Tyr)	c.338G > A (p.Cys113Tyr)
SCADD			*ACADS*		
1	F	Zhuang		c.625G > A (p.Gly209Ser)	c.625G > A (p.Gly209Ser)
2	F	Han		c.268G > A (p.Gly90Ser)	c.337G > A (p.Gly113Arg)^#^
MCADD			*ACADM*		
1	M	Zhuang		c.580A > (p.Asn194Asp)	c.580A > (p.Asn194Asp)
IVA			*IVD*		
1	F	Zhuang		c.214G > A (p.Asp72Asn)	c.1208A > G (p.Tyr403Cys)
2	M	Han		c.548C > T (p.Ala183Val)^#^	c.757A > G (Thr253Ala)^#^
3	M	Zhuang		c.149G > A (p.Arg50His)	c.1199A > G (p.Tyr400Cys)
GA- I			*GCDH*		
1	M	Zhuang		c.532G > A (p.Gly178Arg)	c.532G > A (p.Gly178Arg)
MMA			*MMACHC*		
1	F	Han		c.315C > G (p.Tyr105Ter)	1076bp del^#^
PA			*PCCB*		
1	F	Zhuang		c.1559-4_1559-3insAGAAGCA	c.1594C > T(p.Arg532Cys)
HMGCLD			*HMGCL*		
1	F	Dong		c.252 + 1G > A	c.501C > G (pTyr.167Ter)^#^

*The symbol “#” in superscript represents unreported, and “–” represents no mutation or not checked out.*

**TABLE 6:** Spectrum of IEMs and variants in confirmed cases of high-risk infants.

**IEM**	**Gender**	**Ethnicity**	**Gene**	**Variant 1**	**Variant 2**
CTLN2			*SLC25A13*		
1	F	Han		c.851_854delGTAT	c.851_854delGTAT
2	M	Han		c.851_854delGTAT	c.851_854delGTAT
3	F	Zhuang		c.851_854delGTAT	c.851_854delGTAT
4	M	Zhuang		c.851_854delGTAT	c.851_854delGTAT
OTC			*OTC*		
1	F	Zhuang		IVS7-1G > A	–
2	M	Han		2-7exon del	–
H-TYR			*HPD*		
1	F	Yao		c.93 + 1delG^#^	c.941T > C (p.Ile314Thr)
CPS1			*CPS1*		
1	M	Han		c.1649C > T (p.Thr550Met)	c.858G > C (p.Lys286Asn)
PCD			*SLC22A5*		
1	F	Dong		c.976C > T (p.Gln326Ter)^#^	c.976C > T (p.Gln326Ter)^#^
2	M	Zhuang		c.1411C > T (p.Arg471Cys)	c.1195C > T (p.Arg399Try)
SCADD			*ACADS*		
1	M	Han		c.1148G > A (p.Arg383His)	c.1031A > G (p.Glu344Gly)
2	F	Zhuang		c.320G > A (p.Arg107His)	c.1195C > T (p.Arg399Trp)^#^
CPT2			*CPT2*		
1	F	Han		c.886C > T (p.Arg296Ter)	c.1148T > A (p.Phe383Tyr)
MADD			*ETFA*		
1	M	Han		c.494T > C (Val165Ala)	c.737G > T (p.Gly246Val)^#^
IVA			*IVD*		
1	M	Han		c.1208A > G (p.Tyr403Cys)	c.1208A > G (p.Tyr403Cys)
GA- I			*GCDH*		
1	M	Han		c.532G > A (p.Gly178Arg)	c.655G > A (p.Ala219Thr)
2	M	Zhuang		c.532G > A (p.Gly178Arg)	c.1060G > T (p.Gly354Cys)^#^
MMA			*MMUT*		
1	F	Han		c.103C > T (p.Gln35Ter)	c.346G > A (p.Val116Met)
PA			*PCCB*		
1	F	Han		c.132-134delGACinsAT^#^	–

*The symbol “#” in superscript represents unreported, and “–” represents no mutation or not checked out.*

## Discussion

The incidence of genetic metabolic diseases varies across different countries and regions worldwide. In this study, the overall incidence of genetic metabolic diseases in Liuzhou, Guangxi, was about 1/3,733, which was similar to that reported in other domestic studies. PCD had the highest incidence of all genetic metabolic diseases in newborns in Liuzhou, and ranked first among the fatty acid metabolic disorders, at 1/9,332, which is higher than that reported in other parts of China. The global prevalence rate of PCD is about 1/20,000–1/120,000 (di San Filippo et al., 2008). At present, more than 180 kinds of *SLC22A5* gene mutations have been reported, most of which are missense mutations, followed by non-sense mutations and frameshift mutations, while splice site mutations are relatively rare; the most frequent mutation is exon 1 (Li et al., 2010). The hotspot mutations of the *SLC22A5* gene vary among different races and regions worldwide. R282X mutations are common in Caucasians (Burwinkel et al., 1999; Vaz et al., 1999; Wang et al., 1999), and W132X and W283C mutations are most common in East Asian populations (Koizumi et al., 1999; Nezu et al., 1999; Tang et al., 1999). R254X mutations were found in children with PCD in Taiwan and Shanghai (Tang et al., 2002; Lee et al., 2010). In this study, 11 kinds of 26 mutation sites were detected in newborns and high-risk infants: c.51C > G, c.976C > T, c.1411C > T, c.1195C > T, c.760C > T, c.919delG, c.1400C > G, c.517delC, c.839C > T, c.505C > T, and c.338G > A. Inconsistent with previously reported hotspots of mutations, c.51C > G mutations occurred as often as eight times in 14 children in this study, with a frequency of about 28.6%. At the same time, 2,093 newborns were screened for the *SLC22A5* gene mutation, and 24 cases of reported mutation carriers were detected, of which the c.51C > G mutation occurred as often as 13 times, accounting for 54% of the carriers. The c.51C > G mutation had a high carrying rate in the population of Liuzhou and was the most frequent mutation of the *SLC22A5* gene reported in this study. Primary screening of 119,986 newborns resulted in 2,464 positive tests, from these newborns 2,275 were examined for a second time and 30 positive cases were established. The main reasons for the high false positive rate of tandem mass spectrometry are as follows: The first is the quality of the blood tablets to be tested. For example, whether the blood collection time is standardized, whether the blood collection process is polluted, and the transportation time of blood filter paper is too long. The second is the quality control of the experimental process. Such as reagent preparation, quality control material selection. Finally, the quality audit and interpretation of the experimental results, there may be subjective factors in the interpretation of laboratory results.

PHD had the second highest incidence of all genetic metabolic diseases and ranked first among the amino acid metabolic disorders. In this study, PHD was detected in six newborns, at a rate of 1/18,664. Studies have shown that the incidence of PHD in China is 1/3,000–1/16,000 and incidences are higher in northern China than in southern China. The Liuzhou area of Guangxi is located in the south of China, and the detection rate of PKU is significantly lower than that in other areas in the north of China. So far, more than 800 *PAH* gene mutations have been found in patients with *PAH* deficiency. The *PAH* gene has some hotspot mutations, which vary among different populations. c.728G > A is the most common in China and Korea (Lee et al., 2008; Zhang et al., 2018), the c.1222C > T mutation is the most common in the United States (Kaul et al., 1994), c.1238G > C is common in Japan (Okano et al., 2011), and IVS10-11G > A is the most common in Iran and Spain (Bueno et al., 2013; Rastegar Moghadam et al., 2018). In the present study, eight kinds of 12 mutation sites were found in the PHA gene: c.1174T > A, c.1223G > A, c.208_210del, c.611A > G, c.331C > T, c.728G > A, c.320A > G, and c.721C > T. Further, c.1223G > A was the most common *PAH* mutation site, accounting for 25% of all mutation types, potentially representing a hot spot mutation in this region.

IVA had the third highest incidence of all genetic metabolic diseases and ranked first among organic acidemia disorders, with a detection rate of 1/37,329, which was higher than that reported in previous studies. IVA occurs due to the loss of the functional activity of isovaleryl-CoA dehydrogenase caused by *IVD* gene mutation, resulting in leucine metabolism disorder. The disease was first reported by Tanaka et al. (1966). Significant differences in the incidence of IVA exist among different regions and populations: about 1/250,000 in the United States (Frazier et al., 2006), about 1/365,000 in Taiwan (Lin et al., 2007), and about 1/67,000 in Germany (Ensenauer et al., 2011). Some thermal mutations were found in the *IVD* gene in different regions. The c.932C > T mutation is the main mutation in Germany and the United States (Vockley and Ensenauer, 2006). The c.457-3_2CA > GG mutation is common in Korea (Lee et al., 2007), and the c.149G > C and c.1208A > G mutations are common in Taiwan (Lin et al., 2007). The Chinese scholar Wenjuan Qiu reported a case of IVA with compound heterozygous mutations of the *IVD* gene, c.149G > A and c.466G > C (Qiu et al., 2008). Shiyue Mei reported two cases of IVA and detected compound heterozygous mutations of the *IVD* gene, c.1195G > C and c.466-3_466-2delinsGG, and the homozygous mutation c.1208A > G (Mei et al., 2018). Xiyuan Li reported three cases of children with atypical IVA (Li et al., 2014) in which six mutation types were detected: c.157C > T, c.214G > A, c. 1183C > G, c.1208A > G, c.1039G > A, and c.1076A > G. In this study, five mutation types and eight mutation sites of the *IVD* gene were detected in four children with IVA: c.158G > A, c.214G > A, c.548C > T, c.757A > G, and 1208A > G, of which c.548C > T and c.757A > G were not included in the database. The frequency of the 1208A > G mutation was the highest (appearing four times in this study). In a previous study, high-throughput sequencing was carried out in 2,095 normal newborns in this area, and 10 cases of *IVD* gene carriers were detected, of which as many as five cases were c.1208A > G mutation carriers. At present, no reports are available on the mutation spectrum and hot spots of patients with IVA in China. However, the c.1208A > G mutation has a high frequency in China, according to the *IVD* gene mutation types detected in four children with IVA in this study and other domestic studies. Whether the mutation is a hotspot mutation in the Chinese population or local population needs to be further verified by expanding the sample size.

In this study, the detection rate of SCADD in newborns was about 1/55,993, and the rate in Suzhou, China, was about 1/28,690 (Wang et al., 2019). Zytkovicz reported that the global incidence of SCADD was about 1/25,000–1/45,000 (Zytkovicz et al., 2001). More than 70 types of gene mutations have been reported in ACADS. Gregersen et al. (2008) and Jethva et al. (2008) found that c.625G > A and c.511C > T mutations were dominant in Europe and the United States. The two mutation sites were screened in 694 newborns in the United States, revealing that the c.625G > A mutation accounted for 22% of all detected alleles. C.511C > T accounted for 3%. The incidence of SCADD was significantly lower in the Asian population than in Caucasians, while the carrying rate of the c.625G > A mutation in Hispanics was as high as 30%. In this study, six mutation sites of the ACADS gene were detected in newborns and high-risk infants: c.268G > A, c.320G > A, c.337G > A, c.625G > A, c.1031A > G, c.1148G > A, and c.1195C > T, of which c.337G > A and c.1195C > T were unreported mutation sites; the c.625G > A mutation accounted for 25%, which was consistent with reports from Gregersen et al. (2008) and Jethva et al. (2008). At present, there is still controversy about whether to screen SCADD and some variants of ACADS gene. At the same time, whether the impact of SCADD on the population is the same in different countries or different regions needs to be further observed in the later stage.

One case of glutaric acidemia type I was detected in newborns, and the detection rate was 1/111,986. Two cases were detected in high-risk infants, and the detection rate was 1/3,731. The incidence of glutaric acidemia type I in newborns is about 1/100,000 (Kölker et al., 2011), with ethnic and regional differences. The incidence of glutaric acidemia type I was about 1/130,000 in the United States (Basinger et al., 2006). Of the 129,415 newborns screened, only 2 cases were diagnosed with glutaric acidemia type I in Zhejiang province (Yang et al., 2011). At present, more than 200 *GCDH* gene variants have been reported, with obvious heterogeneity in different regions and populations. The common variation in Pennsylvania is c.1296C > T (Korman et al., 2007), while the hotspot of variation in Taiwan and Hong Kong is IVS10-2A > C (Tang et al., 2000; Shu et al., 2003; Hsieh et al., 2008). At present, no hotspots of variation exist in the Chinese mainland area. Lin et al. (2018) analyzed gene variations in five cases of glutaric aciduria type I (GA-I); c.1244-2A > C mutation frequency was the highest. Three variants of the *GCDH* gene were found by Sanger sequencing: c.532G > A, c. 655G > A, and c.1060G > T, of which c.532G > A appeared four times, which might be a hotspot variation in this area; c.1060G > T variation has not been reported.

In addition, other types of genetic metabolic diseases were also detected, such as hyperhomocysteinemia, MCADD, MMA, propionemia, 3-hydroxy-3-methylglutaric acidemia, ornithine carbamoyltransferase deficiency, H-TYR, CPS1, CPT2, and glutaric acidemia type II. Due to the small number of cases, these diseases were not discussed in this study. In this study, only one mutation was detected in 2 cases of OTC gene and 1 case of PCCB gene, which may be caused by large deletion, deep intronic mutations or promoter mutations. To sum up, after screening newborns and some high-risk infants in this area for genetic metabolic diseases, the detection rate and disease spectrum of some diseases were preliminarily confirmed and some mutation hotspots and new mutations were detected. These mutation hotspots may be potential candidate sites for gene screening and may expand the gene mutation spectrum of genetic metabolic diseases. The findings of this study may have a certain reference value for genetic counseling and prenatal diagnosis of genetic metabolic diseases.

## Data Availability Statement

The original contributions presented in the study are included in the article/supplementary material, further inquiries can be directed to the corresponding author/s.

## Ethics Statement

The studies involving human participants were reviewed and approved by the Ethics Committee of Liuzhou Maternal and Child Health Hospital. Written informed consent to participate in this study was provided by the participants’ legal guardian/next of kin. Written informed consent was obtained from the individual(s), and minor(s)’ legal guardian/next of kin, for the publication of any potentially identifiable images or data included in this article.

## Author Contributions

JT formulated the study, analyzed the data, wrote the manuscript, and designed the tables. DC, RoC, LP, JY, DY, LH, TY, HN, and JW performed screening by MS/MS and collected the data. ReC provided intellectual thoughts, revised the manuscript, and led the project. All authors contributed to the article and approved the submitted version.

## Conflict of Interest

The authors declare that the research was conducted in the absence of any commercial or financial relationships that could be construed as a potential conflict of interest.

## Publisher’s Note

All claims expressed in this article are solely those of the authors and do not necessarily represent those of their affiliated organizations, or those of the publisher, the editors and the reviewers. Any product that may be evaluated in this article, or claim that may be made by its manufacturer, is not guaranteed or endorsed by the publisher.

## Footnotes

1
http://www.hgmd.cf.ac.uk/ac/

2
http://www.ncbi.nlm.nih.gov/

3
http://exac.broadinstitute.org/

4
https://www.internationalgenome.org/

## Abbreviations

CPS1: Carbamyl phosphate synthase 1 deficiency
CPT2: carnitine palmitoyltransferase II deficiency
CTLN2: citrullinemia
GA-I: glutaric aciduria type I
HCY: homocystinuria
HMGCLD: 3-hydroxy-3-methylglutaryl-coenzyme A lyase deficiency
H-TYR: tyrosinemia
IVA: isovaleric acidemia
MADD: multiple acyl-CoA dehydrogenase deficiency
MCADD: medium-chain acyl-CoA dehydrogenase deficiency
MMA: methylmalonic acidemia
OTC: ornithine transcarbamylase deficiency
PA: propionic acidemia
PCD: primary carnitine deficiency
PKU: phenylketonuria
SCADD: short-chain acyl-CoA dehydrogenase deficiency.
